# Successful control of a Methicillin-resistant Staphylococcus aureus outbreak in a neonatal intensive care unit: a retrospective, before-after study

**DOI:** 10.1186/1471-2334-13-440

**Published:** 2013-09-22

**Authors:** Silvia Iacobelli, Benoit Colomb, Francesco Bonsante, Karine Astruc, Cyril Ferdynus, Marie-France Bouthet, Catherine Neuwirth, Ludwig Serge Aho Glélé, Pascal Chavanet, Jean-Bernard Gouyon

**Affiliations:** 1 Néonatologie, Réanimation Néonatale et Pédiatrique, BP 350, 97448, Saint Pierre Cedex, France; 2 Centre d’Etudes Périnatales de l’Océan Indien, CHU La Réunion, France; 3 Service de pédiatrie 2, Hôpital d’enfants, 10, boulevard Maréchal-de-Lattre-de-Tassigny, 21079, Dijon cedex, France; 4 Service d’épidémiologie et d’hygiène hospitalière, Hôpital d’Enfants, CHU, 14 Rue Paul Gaffarel - BP 77908, 21079 Dijon cedex, France; 5 Unité de Soutien Méthodologique, CHU La Réunion, Saint Denis F-97400, France; 6 Laboratory of Bacteriology, University Hospital of Dijon, Plateau technique de Biologie, BP 37013, 21070 Dijon cedex, France; 7 Département d’infectiologie, Hôpital du Bocage, CHU Dijon, BP 77908 F-21079 Dijon, France; 8 Centre d’Epidémiologie des Populations (EA4184), Université de Bourgogne, Dijon, France, 7, Boulevard Jeanne d’Arc, 21000 Dijon, France

**Keywords:** Quality-improvement, Practices, Newborn, Hospital-acquired infection, Community, Endemic, Voice, Bundle

## Abstract

**Background:**

Aim of this study was to provide a detailed description of a Methicillin-resistant Staphylococcus aureus (MRSA) outbreak management strategy in the neonatal intensive care unit of a university hospital.

**Methods:**

This was a retrospective, “before-after” study, over two consecutive 18-month periods. The outbreak management strategy was performed by a multidisciplinary team and included: extensive healthcare workers (HCW) involvement, education, continuous hand-hygiene training and active MRSA colonization surveillance. The actions implemented were identified based on an anonymous, voluntary, reporting system, carried out among all the HCW, and regular audit and feedback were provided to the nursing staff.

The main measured outcome was the rate of MRSA infections before and after the implementation of the outbreak management strategy. Piecewise linear Poisson regression was performed and the model adjusted for confounding variables. The secondary outcome was the rate of laboratory-confirmed bloodstream infections before and after the outbreak management strategy. The rates of MRSA colonization, implementation of proposed actions, observed compliance for hand-hygiene and insertion/care of central lines were also recorded during the second period.

**Results:**

1015 newborns were included. The rate of MRSA infections throughout the two periods fell from 3.5 to 0.7 cases per 1000 patient-days (p=0.0005). The piecewise Poisson regression analysis adjusted for confounding variables showed a significant decrease in the MRSA infection rate after the outbreak management strategy (p=0.046). A significant decrease in positive laboratory confirmed blood cultures was observed over the two periods (160 *vs* 83; p<0.0001). A significant decline in the MRSA colonization rate occurred over the second period (p=0.001); 93% of the proposed actions were implemented. The compliance rate for hand-hygiene and insertion/care of central lines was respectively 95.9% and 62%.

**Conclusions:**

The implementation of multiple, simultaneous, evidence-based management strategies is effective for controlling nosocomial infections. Outbreak management strategies may benefit from tools improving the communication between the institutional and scientific leadership and the ground-level staff. These measures can help to identify individualized solutions addressing specific unit needs.

## Background

Methicillin-resistant *Staphylococcus aureus* (MRSA) infection outbreaks have been widely described in neonatal intensive care units (NICUs) [1-3].

Effective measures for containing these outbreaks have been reported, including the reinforcement of hand-hygiene, staff training, active surveillance, aggressive implementation of contact isolation, cohorting, decolonization and antibiotic stewardship [4-6]. In many studies, the use of “bundle” strategies or simultaneous and multiple practice changes with the aim of eradicating MRSA spreads has been advocated as more successful than the application of single specific measures [4-7].

Usually, the means of implementation is not featured, in particular regarding the most commonly encountered challenges and organizational aspects.

The aim of the present study is to fully illustrate the management strategy to control an outbreak of MRSA infections in the NICU of a university hospital. The identification of actions and the implementation of the multiple established solutions, which have specifically targeted the unit needs, will be described.

## Methods

### Design

This was a retrospective “pre-post” study, evaluating the impact of an outbreak management strategy on the rate of MRSA infections before and after the intervention was implemented. The study was conducted over two consecutive periods: from January 1, 2007 to June 30, 2008 (first period) and from July 1, 2008 to December 31, 2009 (second period).

#### Background, setting and study population

From mid June 2007 to late June 2008, an outbreak of 30 MRSA infections was observed in 30 newborns hospitalized in the III level NICU of the Dijon university children’s hospital.

This is a teaching hospital that provides a range of neonatal care from primary to tertiary level. The 18-bed III level NICU has approximately 350 admissions per year, including both inborn and outborn patients. When this study was performed, the building where the NICU was located was relatively old and the unit beds were distributed in two adjacent areas: a six double room area with 12 beds (the NICU-1) and a three double room area with 6 beds (the NICU-2). NICU-2 was contiguous to the Paediatric Intensive Care Unit (PICU), also admitting children up to the age of 16 (this PICU provided 4 beds for paediatric and 4 beds for cardio-paediatric intensive care).

The NICU room design suffered from a shortage of space, so that the distance between incubators was less than 2 m and the space per incubator less than 5 m^2^, contrary to recommendations [8].

Two separate medical and paramedical teams cared for the newborn babies hospitalized in the NICU-1 and in the NICU-2. In total, 4 paediatricians, 4 residents, 80 children’s nurses, 10 nursing auxiliaries and 10 members of cleaning staff were continuously employed in the two units throughout the study period. Holiday and night work cover of the two units were provided by a senior paediatrician plus a resident, being part of the 14 senior staff physicians and of the 20–25 medical resident staff of the paediatric department. In addition, a wide range of health professionals were sporadically involved in infant care interventions (external consultants, physiotherapists, radiology technicians).

No infant hospitalized in beds for paediatric or cardio-paediatric intensive care had been concerned by the outbreak of MRSA infection, and this study was conducted only among the newborns of the NICU.

### Baseline practices prior to the MRSA outbreak

Sinks and chlorhexidine/alcohol hand antiseptics were available at the entrance of each unit and room. Hand hygiene education for all new staff and family visitors was performed by health care workers (HCW) and self-assessment of risk was promoted via leaflets and posters in the unit. Nurses and physicians changed their uniforms daily; each visitor wore a clean gown and in addition long sleeved gowns were available near the incubator for each direct patient contact. Educational programmes and direct observation for auditing HCW hand hygiene compliance were sporadically performed by auditors external to the unit, without feedback to the staff.

Active surveillance cultures for MRSA colonisation were not systematically carried out for all hospitalized newborns and only long-term patients remaining in the unit were screened on a weekly basis. Infants who were infected or colonized with MRSA were placed in contact isolation. This required the use of barrier precautions such as gloves, gowns and masks for all direct patient contact.

The mean MRSA infection rate calculated over the three years preceding this study was 0.7 cases per 1000 patient-days (95% CI: 0.3-1.1).

### Infection control measures

#### Initial outbreak control measures

After the first 3 cases of infection occurring on June 2007, infection control nurses from the hospital hygiene division performed a sustained HCW information and education session on the importance of scrupulous hand-hygiene, with special emphasis on the utilisation of hydroalcoholic solution before and after every patient contact. Audits of hand-washing performance by ultraviolet light box were regularly carried out. Surface samples were obtained from rooms, incubators, soft toys, monitors and medical devices and all staff were screened for nasal colonization, without identifying any MRSA reservoir or carriage. Soft toys and all items not strictly linked to nursing procedures were nevertheless banned from NICU cots. Reinforced contact isolation for infected patients was implemented.

#### Implementation of the “outbreak management strategy”

Despite these measures, 12 new cases appeared over the next six months, so that, in order to define the general strategy for outbreak control, a steering committee was created. This was a multidisciplinary group including the head of the hospital committee against nosocomial infections, the head of the paediatric department, three of the unit’s paediatricians, the paediatric department senior health manager, the nursing manager of each unit, two nurses for each unit, two physicians from the hospital hygiene division and one from the department of bacteriology.

Starting on January 2008, the steering committee validated an “outbreak management strategy” based on the simultaneous implementation of multiple, evidence based actions. The leading hypothesis of this programme was that the extensive, active engagement of all HCW, added to the continuous monitoring and feedback to staff would have been essential to the success of the project. So, one “operational team” was created in order to carry out the transition from strategy to policy and an anonymous and voluntary “at risk for infection event” and “suggestions for preventing MRSA infection” reporting system, addressed to all HCW, was implemented.

The operational team included: two paediatricians, four quality control nurses (QCNs), one bioengineer, one psychologist, nurses, children’s nursing auxiliaries, health care aids, and cleaning staff, for a total of thirty members, sharing roles and responsibilities for the implementation of the project.

Several mail boxes were placed in readily visible locations all over the units. The reporting forms allowed patient administrative data to be noted along, with a narrative section explaining the event or just describing procedures or policies carried out by HCW or visitors, which were considered at risk for MRSA infection by the reporter. Similarly, suggestions could be proposed for preventing MRSA infection. This step lasted 21 days. Afterwards, the review and analysis process of the reported forms (55 in total) was carried out by the operational team which proposed six strategic priorities, including thirty actions which were retained by the steering committee and put in place from late June 2008 (strategic axis and actions are resumed in Table 1).

**Table 1:** Management strategic axis and actions retained by the steering committee

Quality process implementation	Operational team:
	1. Care quality improvement approach
	2. Identification of 4 “quality control nurses” among the staff nurses
	3. Definition of desired outcomes
	4. Procedure reference tool validation
	5. Random observation of procedure compliance
	6. Procedure compliance evaluation by a computerized feedback tool
	Hospital Hygiene Division:
	7. Hygiene counselling and infections epidemiologic monitoring
	Hospital Executive Board:
	8. Bioengineer consultant recruitment
	All HCW and visitors:
	9. Anonymous “at risk for infection event” declaration
Effective communication	Head of the paediatric department:
	10. Regular reports on MRSA infections management to the hospital executive board, health care branch, department of health quality safety and patient experience
	11. External audit request
	Operational team:
	12. Monthly internal feedback audits
	13. Monthly report display in units
Infections epidemiologic monitoring	Staff nurses:
	14. Routine weekly MRSA screening
	Physicians:
	15. Retrospective and prospective data collection
	Hospital Hygiene Division:
	16. Case–control study to identify risk factors for MRSA infections in hospitalized newborns
	17. Regular environmental and medical device cultures
	Department of bacteriology:
	18. MRSA clinical isolates genotyping
Hand-hygiene, contact precautions, HCW and patients families clothing and flow issues improvement	Hospital Hygiene Division:
	19. Intensive HCW, families and visitors information and training
	20. HCW, families and visitors work clothing + protective clothing implementation
	21. Reinforced barrier precautions for MRSA colonization
	22. Posted isolation cards
Standardisation of procedures for the insertion and the continuous care of peripherally inserted central venous catheters, care of invasive medical devices	Staff nurses:
	23. Central venous lines insertion and care checklist
	24. Invasive medical devices care checklist
Units cleaning	Cleaning staff:
	25. Cleaning procedure implementation
	26. Cleaning procedure assessment
	27. Cleaning checklist
	28. Cleaning staff overwork lowering (2 recruitments)
	29. Room cleaning intensification (3 daily cleanings versus 1)
	30. Standardized disinfection of external medical devices

Together with the above actions the following interventions were implemented:

1) Comprehensive information on mechanisms and prevention of nosocomial infections was given by the staff of the hospital hygiene division to the department’s HCW.

2) Four QCNs were dedicated to the observation of hand-hygiene compliance and adequate application of the retained actions. These nurses shared their workload between quality care and patient care. Data were collected in a structured form on a PC pocket during observation and were analysed each month by an independent bioengineer.

3) A monthly staff meeting of the operational team was organized, to point out the strategic priorities and to present data on hygiene compliance, rate of MRSA infections and MRSA colonization. All the above mentioned external HC providers were invited to these meetings.

4) A monthly feed-back of data and advances was given to all the HCW by reports displayed in visible locations all over the units.

5) Intensive HCW information and training on hand-hygiene was continuously performed by the infection control nurses from the hospital hygiene division throughout the period.

**Figure 1:**
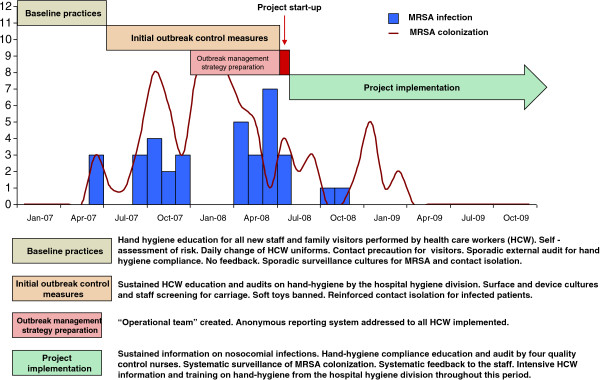
**Timing of all the interventions during the study period in relation to Methicillin-resistant *Staphylococcus aureus *(MRSA) cases.**

Figure 1 shows the timing of all the interventions during the study period in relation to MRSA cases.

### Data sources

For the purpose of this work, information was retrieved from the regional database of the Burgundy perinatal network [9,10], from medical charts, and from the specifically created procedure observation files.

Data collected included infant demographic, gestational and clinical variables, comprising perinatal risk factors for hospital-acquired infection (HAI) : clinical features, type and duration of mechanical ventilation (MV), duration of central venous lines (CVL), septic episodes and outcome, culture and laboratory results, antibiotic exposure, length of stay and bed occupancy rate. The average number of nurses per patient (nurse-to-patient ratio) was calculated for each day of the study period.

### Outcome measures and definitions

The main outcome chosen for the analysis was the rate of MRSA infection per 1000 patient-days before and after the implementation of the outbreak management strategy.

In accordance with the standard definition of HAI from the Center for Disease Control (CDC) [11] any infant with clinical isolate of MRSA, who was receiving antimicrobial therapy, was categorized as experiencing an episode of infection of MRSA.

The secondary outcomes were: laboratory-confirmed bloodstream infection rates from *Staphylococcus sp.* other than MRSA and from “other Gram positive” and Gram negative pathogens. These were defined according to the above reference from the CDC [11].

The following quality indicators were also evaluated over the second period: rate of MRSA colonization, implementation of proposed actions and observed compliance for hand-hygiene and insertion/care of CVL.

Starting in July 2008, weekly screening for MRSA colonization was performed by swabbing the anterior nares of all neonates present in the units and for all newly admitted newborns. Cultures were performed on MRSA selective medium (Métistaph 2, AES). Bacteriological analysis of clinical samples was performed as usual, including a culture on Trypcase soy agar + 5% sheep blood.

Infants colonized or infected by MRSA underwent contact isolation (without cohorting) until the detection of a double negative swab culture.

The rate of compliance for hand-hygiene was defined as the number of times that hands were disinfected, compared with the number of situations where it was necessary, according to the reference [12]. The rate of compliance for insertion/care of CVL was the proportion of procedures considered correct, according to the unit guidelines for CVL.

These were evaluated for the duration of the second period by random weekly observations from the QCN using specific check-list files for each procedure.

Data on the consumption of hydroalcoholic solution during the study period were also analysed. This was expressed in L/1000 patient-days.

### Statistical analysis

Data are presented as mean and standard deviation (SD) for continuous variables and as numbers and proportions (%) for categorical ones. Comparisons between groups were performed using χ^2^-test or Fisher’s exact test for categorical variables;

Patients of the first period were compared with the patients of the second period taking into account demographic, clinical characteristics and perinatal risk factors for MRSA infection.

A piecewise linear Poisson regression was performed to compare MRSA infection rates before and after the outbreak management strategy implementation. The model was adjusted for potential confounding variables (caesarean delivery rate, duration of MV and CVL, use of antibiotics, occupancy rate and nurse-to-patient ratio).

Statistical analyses were performed by R 2.12 (The R Foundation for Statistical Computing, Free Software Foundation Inc., Boston, MA, USA) and SAS 9.2 (SAS Institute, Cary, NC, USA).

### Ethics and consent

In Burgundy clinical data on the entire newborn population are collected by the regional database of the Burgundy Perinatal Network. This database was set up with the approval of the National Committee for data protection (CNIL registration number 98003718) and prospectively records clinical events for mothers and infants between birth and hospital discharge. On behalf of the Burgundy Perinatal Network, JBG, president of the Network, has the permission to access the database.

Due to the retrospective character of this study, approval of the research ethics committee in our hospital was not needed. According to French legislation, written parental consent was not needed for this retrospective study.

## Results

### Study population

A total of 1015 neonates were admitted to the NICU during the study period and included in the analysis. In the first period 510 infants were hospitalized and 505 in the second, for a total of 16 163 patient-days (8567 and 7596 patient-days for, respectively, the first and the second period). Table 2 shows demographic data and perinatal risk factors for HAI in the two groups.

**Table 2:** Demographic data and perinatal risk factors for hospital acquired infections in the study population

	**1**^**st **^**period**	**2**^**nd **^**period**	**p-value**
	**Infants = 510**	**Infants = 505**	
Gestational age (weeks)	32.7 ± 4.2	33.0 ± 4.5	0.31
< 34 (%)	58.6	55.6	0.17
34 - 36 (%)	20.0	18.0	
> 36 (%)	21.4	26.4	
Birth weight (grams)	1934 ± 864	2022 ± 942	0.12
Sex male (%)	56.1	54.9	0.69
Antenatal steroids (%)	56.7	54.1	0.37
Clinical chorioamnionitis (%)	13.1	10.1	0.13
Apgar score ≤ 3 at 1 min (%)	12.7	12.1	0.17
Inborn (%)	71.8	71.3	0.87
Caesarean delivery (%)	62.3	54.3	0.0089
Use of surfactant (at last once) (%)	55.7	43.2	<0.001
Respiratory Distress Syndrome (%)	79.8	78.2	0.53
BPD @ 28 days (%)	17.6	19.4	0.47
IVH 3°- 4° (%)	3.1	2.4	0.33
NEC all stages (%)	3.3	2.8	0.60
Malformations (%)	15.9	19.2	0.16
Mean duration of stay in III level NICU (days)	16.8 ± 21.2	15.0 ± 19.6	0.08
Mean total duration of stay (days)	47.0 ± 35.3	48.4 ± 40.3	0.94
Death (%)	5.9	5.4	0.71
IMV (% of infants per day)	0.23 ± 0.09	0.18 ± 0.09	0.0001
NCPAP (% of infants per day)	0.49 ± 0.11	0.42 ± 0.12	0.0001
CVL (% of infants per day)	0.36 ± 0.13	0.34 ± 0.11	0.15
Means number of antibiotics (per infant per day)	0.48 ± 0.22	0.53 ± 0.23	0.001
Occupancy rate (per day)	0.87 ± 0.09	0.77 ± 0.11	< 0.0001
Nurse to patient ratio (per day)	1.35 ± 0.19	1.53 ± 0.30	< 0.0001

*Abbreviations: BPD* Bronchopulmonary Dysplasia, *IVH* Intraventricular Haemorrhage, *NEC* Necrotizing Enterocolitis, *NICU* Neonatal Intensive Care Unit, *IMV* Invasive Mechanical Ventilation, *NCPAP* Nasal Continuous Positive Air Pressure, *CVC* Central Venous Lines.

### Main outcome (MRSA infection incidence)

Thirty MRSA infections occurred in the first period and five in the second. All infants had MRSA isolated from blood culture; 14 infants developed clinical sepsis, 12 pneumonia, 4 meningitis, 3 endocarditis proven by echocardiography, 1 bone and joint infection, and 1 necrotizing enterocolitis. Three infants died.

MRSA infection incidence was significantly higher in the first period with 3.5 cases per 1000 patient-days (95% confidence interval [CI]: 2.4-5.0) versus the second, with 0.7 cases per 1000 patient-days (95% CI: 0.3-1.6) (p=0.0005).

**Figure 2:**
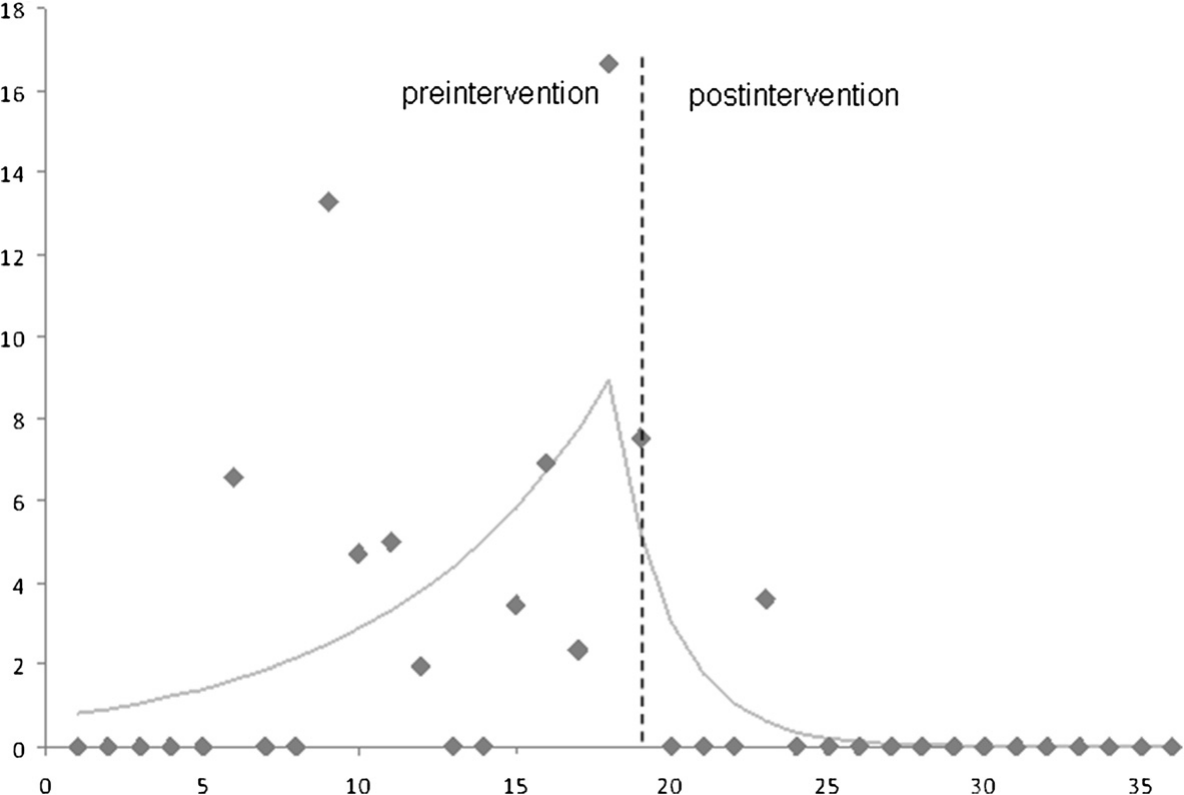
**Rates of Methicillin-resistant *Staphylococcus aureus *(MRSA) infections before and after the outbreak management strategy implemented at month 19.** On the *x* axis, time (months); on the *y* axis, rate of MRSA infection (number for 1000 patient-days).

The piecewise Poisson regression analysis adjusted for confounding variables showed a significant decrease in the MRSA infection rate after the implementation of the outbreak management strategy (p=0.046) (Figure 2).

### Secondary outcomes

With the exception of other Gram positive pathogens, the rate of laboratory-confirmed bloodstream infections was significantly lower during the second period (data shown in Table 3).

**Table 3:** Laboratory-confirmed bloodstream infections (total cases) throughout the study period

	**1**^**st **^**period**	**2**^**nd **^**period**	**p-value**
	**Infants = 510**	**Infants = 505**	
Positive laboratory-confirmed blood culture	160	83	< 0.0001
MRSA	25	5	0.0002
*Staphylococcus sp. other than S. aureus*	115	70	0.0003
“Other gram positive” pathogens	9	5	0.29
Gram negative pathogens	11	3	0.03

### Other quality indicators

**Figure 3:**
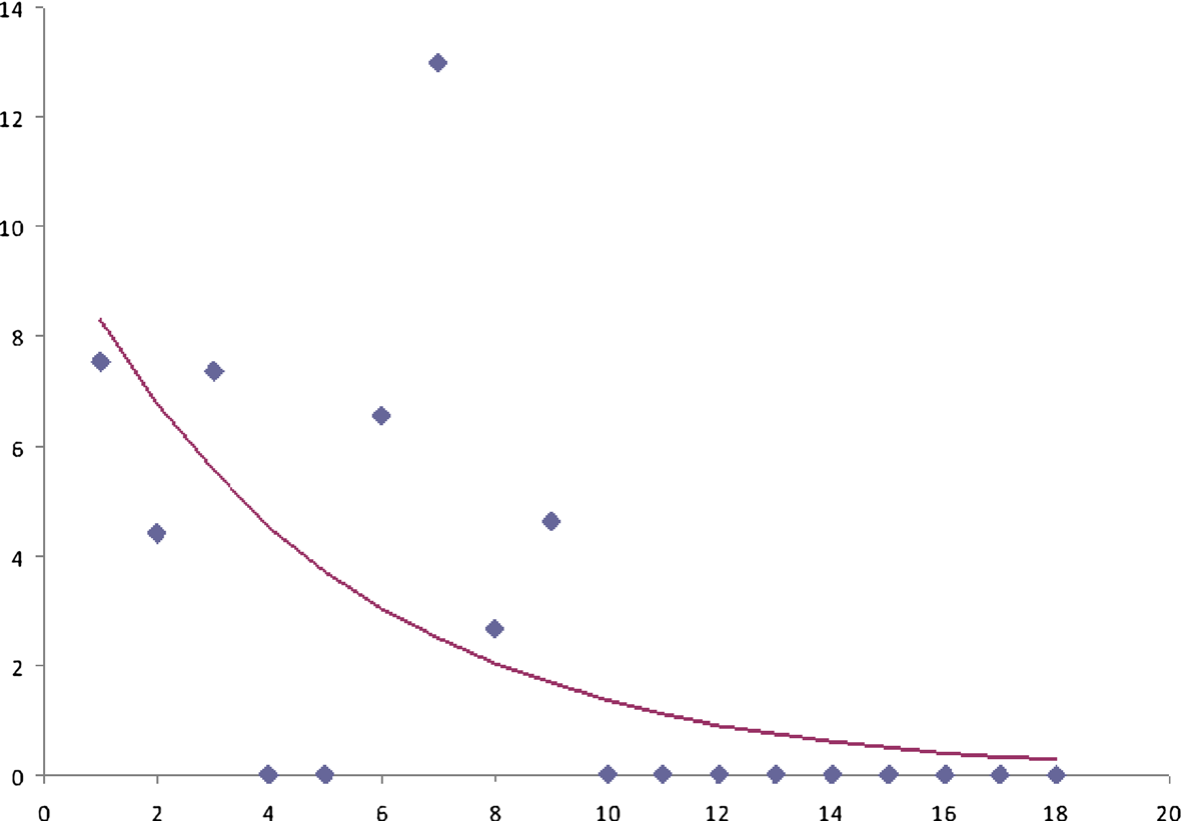
**Decline of Methicillin-resistant *Staphylococcus aureus *(MRSA) colonization rate over the second period of the study.** On the *x* axis, time (months); on the *y* axis, rate of MRSA colonization (number for 1000 patient-days).

The rate of MRSA colonization declined significantly over the second period (p=0.001) (Figure 3).

The rate of retained actions implementation was 93% as only two retained actions were not implemented: the case–control study to identify risk factors for MRSA infections and the MRSA clinical isolates genotyping (only performed on certain patients). Over the second period the compliance rate for hand-hygiene and insertion/care of CVL was 95.9% and 62%, for a total of 1722 and 75 observations, respectively.

The hydroalcoholic solution consumption calculated on period basis was 137.61 and 227.06 L/patient-days in the first and the second period respectively.

## Discussion

Our study shows that a multidisciplinary quality improvement approach can implement a sustainable prevention strategy, resulting in the reduction of invasive MRSA infections in NICU. In our unit, this also led to a significant decrease in other bloodstream nosocomial infections, a goal which was not primarily targeted by the prevention policy.

The importance of a multidisciplinary approach, along with a bundle strategy for the prevention and control of MRSA spread in NICU, is widely recognised and it has been reported elsewhere [4-7]. While taking into account these different papers, it is interesting to note that similar adopted measures are not universally successful and that in some cases, as in the study of Lepelletier et al. [5], several consecutive actions had to be added, in order to control the MRSA outbreak. One report from Haley and colleagues [13] has successfully demonstrated how identical measures applied to eradicate endemic MRSA in their NICU had a different impact over time, according to changing local factors (overcrowding and understaffing).

Actually, clinical reviews concerning adult patients [14] show that, even if guidelines and recommendations for prevention and control of MRSA are similar worldwide, this problem has very different prevalence from country to country, thus suggesting that relevant differences must exist depending on the method used to implement the best practices.

The strength of our study, in relation to previous investigations in this field, was to describe in detail the step-by-step process which allowed us to convert the common knowledge about preventive and control strategies into an effective change of behaviours and organization. This process is often difficult, as it must take into account the extensive understanding of specific difficulties, sometimes pertaining to local factors affecting HCW attitudes.

The innovative tools of our approach were: 1) the anonymous reporting system implementation and 2) the institution of the operational team.

Reporting systems, recommended by the Institute of Medicine [15] for identifying and addressing medical errors, have been increasingly used in neonatology to prevent and manage iatrogenic events [16,17], but in the context of this study the anonymous mail box was mainly used to propose positive inputs and organizational suggestions for the project, as in private companies [18].

The key benefit of the operational team was that this was very representative of the multiple categories of health providers devoted to the continuous care of newborn infants in our units. Its use of feedback communication systems and its accountability for elaborating written procedures facilitated the acceptance of the multiple changes required for the success of the outbreak ma-nagement strategy. This group represented a credible voice, permitting the useful communication between the institutional and scientific leadership and the ground-level staff, and it also allowed an effective exchange between the two distinct teams caring for newborns in NICU 1 and NICU 2.

The implication of the cleaning staff members in the project was also remarkable and we believe that it played an important role in the improvement of the local hygiene conditions of our obsolescent structures.

Our study has several limitations. First, we recognize that the retrospective collection of data does not represent the most rigorous approach to evaluate clinical management or quality improvement interventions. In particular, the lack of a prospective reporting system for some indicators during the first part of the study is a possible bias that limits the interpretation of the results.

The incidence of MRSA infections declined substantially after the intervention, but we acknowledge that the study methodology make it impossible to determine whether the decline resulted from the natural history of the outbreak or a delayed response to the initial outbreak control measures. Moreover, the uncontrolled, before-after design does not allow us to determine which, or even if, any of the bundle interventions contributed to the reduction of MRSA infection rate, and this is the major limitation of this study.

Secondly, our results cannot be automatically generalized, as they represent the consequence of an implementation process which was appositely tailored upon our unit specificities.

Finally, a great effort of inter-institutional discussion and inter-professional collaboration was required by our strategy and regrettably, the economic costs of this effort were not estimated in our study and we trust that this outcome would have been very interesting for the readers.

## Conclusion

We believe that our report has interesting implications for clinicians and policymakers, as it proves that the effectiveness of projects for improving patient care may benefit from structured tools improving the communication between the institutional and scientific leadership and the health givers more directly involved in patient care. The sharing of ideas and responsibilities is useful for the identification of local specificities and individualized solutions.

This work thus represents a useful contribution to the comprehensive evaluation of factors influencing the success of quality implementation practices in the vulnerable population of sick newborn infants.

## Abbreviations

CDC: Center from disease control; CVL: Central venous lines; HAI: Hospital-acquired infection; HCW: Healthcare workers; MRSA: Methicillin-resistant *Staphylococcus aureus*; MV: Mechanical ventilation; NICU: Neonatal intensive care unit; PICU: Paediatric intensive care unit; QCNs: Quality control nurses.

## Competing interest

The authors declared that they have no competing interests.

## Authors’ contribution

SI conceptualized and designed the study, carried out the interpretation of the data and wrote the paper. BC conceptualized and designed the study, performed the acquisition of the data and participated to the interpretation of the data. FB participated to the draft of the initial manuscript, provided a substantial contribution to data collection and analysis. KA participated to the conception of the study, the acquisition and the interpretation of the data. CF performed the statistical analyses. MFB performed the monthly analysis of data during the implementation project. CN was responsible for the bacteriological analyses. SA participated to the design of the management strategy and implementation. PC conceived of the study, and participated in its design and coordination. JBG conceptualized and designed the study, critically reviewed and revised the manuscript drafts. All the authors read and approved the final manuscript as submitted.

## Pre-publication history

The pre-publication history for this paper can be accessed here:

http://www.biomedcentral.com/1471-2334/13/440/prepub
